# Active Transport to School and School Neighbourhood Built Environment across Urbanisation Settings in Otago, New Zealand

**DOI:** 10.3390/ijerph17239013

**Published:** 2020-12-03

**Authors:** Mohammad Lutfur Rahman, Tessa Pocock, Antoni Moore, Sandra Mandic

**Affiliations:** 1 Active Living Laboratory, School of Physical Education, Sport and Exercise Sciences, University of Otago, Dunedin 9054, New Zealand; sandy07a@gmail.com; 2 Faculty of Medical and Health Sciences, School of Nursing, The University of Auckland, Auckland 1142, New Zealand; t.pocock@auckland.ac.nz; 3 School of Surveying, University of Otago, Dunedin 9054, New Zealand; tony.moore@otago.ac.nz; 4 Centre for Sustainability, University of Otago, Dunedin 9054, New Zealand; 5 Faculty of Health and Environmental Sciences, School of Sport and Recreation, Auckland University of Technology, Auckland 1142, New Zealand

**Keywords:** active transport, school neighbourhood, built environment, safety, walking, cycling, adolescents

## Abstract

The school neighbourhood built environment (BE) can facilitate active transport to school (ATS) in adolescents. Most previous studies examining ATS were conducted in large urban centres and focused on BE of home neighbourhoods. This study examined correlations between school-level ATS rates among adolescents, objectively measured school neighbourhood BE features, and adolescents’ perceptions of the school route across different urbanisation settings. Adolescents (n = 1260; 15.2 ± 1.4 years; 43.6% male) were recruited from 23 high schools located in large, medium, and small urban areas, and rural settings in Otago, New Zealand. Adolescents completed an online survey. School neighbourhood BE features were analysed using Geographic Information Systems. School neighbourhood intersection density, residential density and walkability index were higher in large urban areas compared to other urbanisation settings. School-level ATS rates (mean 38.1%; range: 27.8%–43.9%) were negatively correlated with school neighbourhood intersection density (r = −0.58), residential density (r = −0.60), and walkability index (r = −0.64; all *p* < 0.01). School-level ATS rates were also negatively associated with adolescents’ perceived safety concerns for walking (r = −0.76) and cycling (r = −0.78) to school, high traffic volume (r = −0.82), and presence of dangerous intersections (r = −0.75; all *p* < 0.01). Future initiatives to encourage ATS should focus on school neighbourhood BE features and minimise adolescents’ traffic safety related concerns.

## 1. Introduction

Physical activity (PA) contributes to the physical and mental well-being of adolescents [1,2]. However, PA declines during adolescence [3,4]. Approximately 80% of adolescents fail to meet the PA guidelines to accumulate ≥60 min of moderate-to-vigorous PA per day [5]. Decreasing levels of PA among adolescents is now a major public health concern [6,7]. Active transport to school (ATS) (i.e., walking and cycling) is an inexpensive and convenient way to incorporate PA into the everyday lives of adolescents [8,9], and could be a promising strategy to contribute towards adolescents’ daily moderate-to-vigorous PA [10]. The rates of ATS among adolescents vary across countries [4] but are low and declining in developed countries [2,11]. In New Zealand, less than one third of adolescents use ATS [12].

In addition to home-to-school distance, which is the strongest predictor of ATS [13,14], objectively measured and perceived built environment (BE) features of the school and home neighbourhoods are also associated with ATS in adolescents. Most previous studies have examined the characteristics of home neighbourhoods [15,16,17,18,19,20] with only a limited number of studies focusing on school neighbourhoods [21,22]. Objectively measured features of the school neighbourhood BE in previous studies included residential density, intersection density, land use mix, and neighbourhood walkability [21,22,23]. Commonly examined perceptions of school neighbourhood BE features included perceptions of aesthetics, quality of walking and cycling infrastructure, and BE features related to traffic and personal safety [21,22]. BE features related to traffic safety (e.g., dangerous intersections and/or crossings, high traffic volume and/or speed, absence of traffic signals, and absence of footpaths and/or cycle paths) and perceived personal safety (e.g., presence of strangers, local crime, surveillance, and poor street lighting) play a role in influencing adolescents’ and their parents’ perceptions of the safety of walking and cycling home-to-school routes [19,24,25].

The presence of different school neighbourhood BE features and their associations with adolescents’ ATS rates may vary across different urbanisation settings. For example, BE features frequently associated with the presence of footpaths and cycle paths are more common in large urban centres compared to rural settings [22]. Most previous studies have examined the association between school neighbourhood BE features and adolescents’ ATS in large urban areas [21,23], whereas few studies focused on rural areas [22]. These studies found that higher residential density and school neighbourhood walkability were positively associated with adolescents’ ATS in both urban and rural areas [22,23]. Furthermore, higher intersection density in school neighbourhoods was associated with higher rates of ATS in rural areas in the United States [22]. In contrast, adolescents’ traffic and personal safety concerns within the school neighbourhood and the presence of intersections along the route to school were negatively associated with ATS rates in urban areas [21]. However, several studies did not find significant associations between adolescents’ ATS rates and school neighbourhood BE features [22,23]. Whether similar school neighbourhood BE features also correlate with adolescents’ ATS rates in medium and small urban areas remains unknown.

With the exception of several recent studies [21,26,27], most previous studies that have examined the associations between the school neighbourhood BE and ATS rates in adolescents did not limit the study sample to adolescents who lived within walking and/or cycling distance to their school. A recent New Zealand study showed that parental perceptions of barriers to adolescents’ walking and cycling to school varied by home-to-school distance [27]. As the distance to school increased from walkable to cyclable and beyond cyclable, parents of adolescents reported declining levels of social support, perceived more environmental barriers and had greater safety concerns with walking to school and, less consistently, with cycling to school [27]. The aim of this study was to examine the associations between school-level ATS rates among adolescents who lived within a reasonable cycling distance (≤4 km) to school, and objective and perceived measures of school neighbourhood BE features across different urbanisation settings in Otago, New Zealand.

## 2. Materials and Methods

### 2.1. Participants

**Figure 1:**
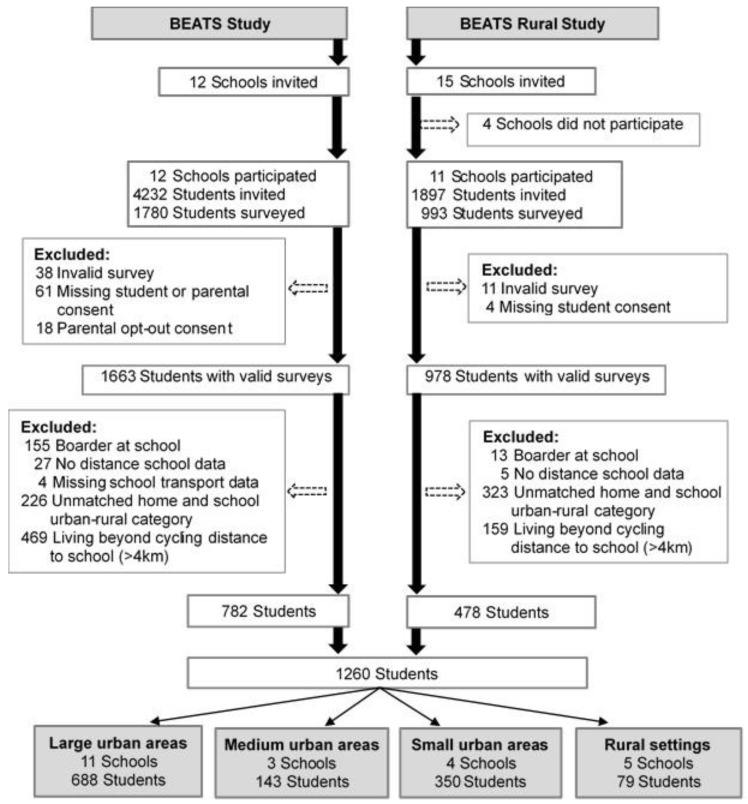
Flow chart of study sample selection. BEATS (Built Environment and Active Transport to School).

This study used secondary data from 23 (out of 27) high schools located in the Otago region; New Zealand collected as part of the Built Environment and Active Transport to School (BEATS) Study in 2014/15 (12 schools; 1780 adolescent participants; 42% adolescent recruitment rate) [28] and the BEATS Rural Study in 2018 (11 schools; 993 adolescent participants; 52% adolescent recruitment rate). Based on classification from Statistics New Zealand, urbanisation settings were categorized into major urban centres (population ≥100,000), large urban areas (≥30,000 to 99,999 residents), medium urban areas (10,000–29,999 residents), small urban areas (≥1000 to 9999 residents) and rural settings (<1000 residents) [29]. Participating schools were located in large (n = 11), medium (n = 3), and small urban areas (n = 4), and rural settings (n = 5). Using a previously published threshold of ≤2.25 km as a reasonable walking distance to school [21] and ≤4 km as reasonable cycling distance to school [30], adolescents were classified into two groups based on home-to-school distance: within walking distance to school (≤2.25 km) and beyond walking but within cycling distance to school (≤2.25 km to ≤4.0 km). Adolescents living >4.0 km from their school were excluded from the analysis. Out of 2825 adolescents (13–18 years of age) who participated in the BEATS and BEATS Rural Studies, data collected from 1260 adolescents who lived within 4 km from their school and attended schools in the same type of urbanisation settings were included in this analysis (Figure 1).

### 2.2. Procedures

School and adolescent recruitment and survey data collection were completed using the BEATS research methodology, as described elsewhere [28]. Briefly, invited adolescents from participating schools received study information packages for themselves and their parents, one to three weeks prior to data collection. Each adolescent signed paper consent. Parental opt-in and opt-out consents (based on schools’ preference) were used in the BEATS Study, whereas no parental consent was required for the BEATS Rural Study. The study protocols were approved by the University of Otago Human Ethics Committee (BEATS: 13/203, July 2013; BEATS Rural: 17/178, November 2017).

### 2.3. Assessment

#### 2.3.1. Questionnaire

In both studies, adolescents completed a 30–40-min online BEATS Student Survey [28] during class time and under the supervision of research staff. The survey included items related to socio-demographic characteristics, travel to school, and adolescents’ perceptions of walking and cycling to school and of their route(s) from home to school.

Socio-demographic data included the date of birth, gender, ethnicity, availability of bicycles and vehicles at home, and home address [28]. Age at survey was calculated from the date of birth. Home address was used to determine neighbourhood deprivation based on the New Zealand Index of Deprivation (data collected on a scale of 1 to 10; 1 = least deprived to 10 = most deprived, and subsequently categorised into quintiles) [31]. Distance from home to school was determined using Geographic Information Systems (GIS) (ArcGIS 10.6.1 software; Esri, Redlands, CA, USA) shortest-path network analysis based on geocoded home addresses and a connected street network [28].

Adolescents self-reported the frequency of using different modes of transport to school (such as, being a passenger in a car, driving a car, travelling by school bus or public transport, on foot, by bicycle or other modes and mixed modes) [32]. Based on their dominant modes of transport to school “most of the time” or “all of the time”, adolescents were classified into users of active transport, motorised transport or mixed active and motorised transport [26]. School-level ATS rates were calculated based on the proportion of adolescents who used active transport and lived within reasonable cycling distance to school and expressed as a continuous variable.

Adolescents’ perceptions of walking and cycling to school and the route to school were assessed using items described elsewhere [28]. Items related to adolescents’ and their parents’ perceptions (as reported by adolescents) of the safety of walking and cycling to school, perceptions of traffic volume and dangerous crossings, and availability of footpaths or cycle paths along the school routes were used in this analysis. Adolescents’ perceptions were assessed using a 4-point Likert scale with response categories “strongly disagree” (1), “somewhat disagree” (2), somewhat agree” (3), and “strongly agree” (4) and analysed as continuous variables. School-level variables for adolescents’ perceptions were created by calculating the mean score of agreement with for each statement (range 1 to 4) for each school.

#### 2.3.2. Objectively-Measured Built Environment Features in the School Neighbourhood

In the present study, school neighbourhoods were defined using both 0.5 km and 1.0 km street-network buffer sizes around individual schools as both of those buffer sizes were used in previous research [33,34] (i.e., a neighbourhood defined by measuring 0.5 km and 1.0 km out from the school along with the road network in all directions). School neighbourhood BE features included intersection density (per km^2^), residential density (per km^2^), land use mix, and walkability index and were calculated for each school within each street-network buffer using previously described procedures [28,35].

Briefly, intersection density was calculated as a count of intersections within each predefined street-network buffer zones around each school [35]. Residential density was calculated as the ratio of residential address points to the land area for each street-network buffer zone around individual schools. Land use mix was calculated using an entropy score inside each street-network buffer for each school [36]. Due to inconsistent categories of land use data collected from respective district councils in the Otago region of New Zealand, land uses in this study were harmonised into four fundamental land use categories (residential, commercial, industrial, and rural) that each district council had in common and were used to calculate mean land use entropy. The missing land uses (for 11 schools) were digitized and adjusted using ArcGIS 10.6.1 software (Esri, Redlands, CA, USA) and Open Street Map (OpenStreetMap Foundation, Cambridge, UK). The land use mix for each school neighbourhood was calculated using the mean land use entropy equation (Equation (1)) [35].

(1) $$\left\{\sum_{k\;}\left[\sum_{j}P_{jk}ln\left(p_{jk}\right)\right]|ln\left(J\right)\right\}/K$$

where *P_jk_* = Proportion of land use category *j* within a half-mile radius of the developed area surrounding hectare grid cell *k*; *j* = number of land use categories; and *K* = number of actively developed hectares in the tract. The mean land use mix entropy was estimated using line-generated service areas buffered at 95 m from the road centreline. The land use mix scores were coded between 0 (the land use mix around each school is of a single class) and 1 (the land use mix around each school is evenly distributed among all land use classes) [35]. The walkability index for each school was calculated as a sum of standardized z-scores for intersection density, residential density and land use mix values [37] using Equation (2).

Walkability Index = (z score of intersection density) + (z score of residential density) + (z score of land use mix)(2)

### 2.4. Data Analysis

School-level data and adolescents’ sociodemographic characteristics were analysed using descriptive statistics. Comparison across geographical settings was performed using a Chi-square test for categorical variables and one-way ANOVA for continuous variables with Scheffe posthoc multiple comparisons or Tamahane’s T2 when the assumption of homogeneity of variance was violated. Correlations were examined using a Pearson product-moment correlation. Data was analysed using IBM SPSS statistics software (version 24.0, IBM, Armonk, NY, USA). *p*-value of <0.05 indicated statistical significance.

## 3. Results

### 3.1. School and Adolescents’ Characteristics

Characteristics of participating schools located across urbanisation settings are presented in Table 1. Mean school-level rates of ATS among adolescents who lived within 4 km from their school was 48.3% (Table 1; median 48.9%). The school-level ATS rates were significantly lower in large urban area schools (41.8%) compared to rural schools (59.5%) (Table 1).

Characteristics of adolescent participants are presented in Table 2. Among 1260 adolescents included in this analysis (age: 15.2 ± 1.4 years), 43.6% were males, 71.4% were New Zealand European, 68.3% lived in households with two or more vehicles and 57.1% had two or more bicycles that they could use to get to school (Table 2). The mean distance to school was 1.8±1.0 km, with significant differences across urbanisation settings (Table 2). Overall, 68.7% of adolescents lived within walking distance to school, and 31.3% lived beyond walking but within cycling distance to school (Table 1). Among adolescents living within 4 km from their schools, a significantly higher proportion of adolescents attending rural schools lived within walking distance to school compared to their counterparts in large urban area schools (Table 1). The proportion of adolescents who lived beyond walking distance but within cycling distance to school ranged from nearly half the adolescents in large urban area schools to less than 10% in rural schools (Table 1).

### 3.2. Objectively-Measured School Neighbourhood Built Environment Features

School neighbourhood BE features varied across urbanisation settings with trends for decreasing mean residential density, intersection density and walkability in school neighbourhoods when moving from more urbanised to less urbanised school locations. Mean school neighbourhood residential density was higher in large urban area schools compared to small urban area schools (1.0 km street-network buffer) and rural schools (0.5 km and 1 km street-network buffers) (Table 3). Similar findings were observed for school neighbourhood intersection density within a 1 km street-network buffer with higher intersection density present in school neighbourhoods of large urban area schools compared to small urban area and rural schools. Mean school neighbourhood BE walkability index was significantly higher in large urban area schools compared to rural schools. Mean land use mix in school neighbourhoods did not differ significantly across urbanisation settings. School-level ATS rates were negatively correlated with school neighbourhood BE intersection density, residential density, and walkability index across both 0.5 km and 1.0 km street-network buffers. A weak but statistically significant positive correlation was found between school-level ATS rates and land use mix (Table 4).

### 3.3. Adolescents’ Perceptions of Their Route to School

Using a school-level data analysis, adolescents attending schools in large urban areas more frequently reported concerns of high traffic volume and number of dangerous intersections along their school route, compared to their peers who attended rural schools (Table 3). Adolescents attending large urban area schools also reported higher levels of their own and their parents’ concerns about the safety of walking and cycling to school compared to adolescents from small urban area schools (cycling concerns only) and rural schools (both walking and cycling concerns). (Table 3). No significant differences were found between adolescents’ perceptions of the availability of footpaths and cycle paths along school routes across the four urbanisation settings examined in this study.

Overall, school-level ATS rates (as reported by adolescents) were negatively correlated with adolescents’ reports of high traffic volume and presence of dangerous intersections along the school routes as well as with adolescents’ and their parents’ concerns about the safety of walking and cycling to school (Table 4). In this study, there were no significant correlations between adolescents’ perceptions of the presence or absence of footpaths or cycle paths and school-level ATS rates.

## 4. Discussion

This study examined associations between school-level ATS rates, objectively-measured school neighbourhood BE features, and perceptions of the school route among adolescents living within 4 km of their school in different urbanisation settings in the Otago region, New Zealand. The key findings include: (1) objectively-measured school neighbourhood BE features were significantly different across urbanisation settings with most significant differences observed between schools located in large urban areas versus those in small urban areas and rural settings; (2) school-level ATS rates were negatively correlated with school neighbourhood BE characteristics including intersection density, residential density, and school neighbourhood walkability; (3) adolescents’ perceptions of traffic volume, and presence of dangerous intersections along the school routes were negatively correlated with ATS rates; and (4) school-level ATS rates were also negatively correlated with adolescents’ perceptions of safety concerns related to walking or cycling to school. Taken together, these findings indicate that school neighbourhood BE features and adolescents’ perceptions of the school route were associated with the use of ATS among adolescents who live within 4 km from their school.

Objectively-measured school neighbourhood BE features were significantly different across urbanisation settings. In this study, the 0.5 km street-network buffer captured BE features within the immediate school neighbourhood environment, while the extended 1.0 km street-network buffer gave a more complete picture of the BE between adolescents’ home and school. Objectively-measured school neighbourhood residential density was significantly different in large urban areas versus rural settings in 0.5 km of street-network buffer around individual schools. On the other hand, school neighbourhood intersection density, residential density, school neighbourhood walkability, and adolescents’ perceptions of safety concerns along the school routes were varied significantly across urbanisation settings when data were analysed using 1.0 km of street-network buffer around individual schools. Most significant differences were observed between schools located in a large urban area versus those in rural settings, with some differences also observed between schools in large versus small urban areas. These findings are not directly comparable to findings from previous studies due to the lack of studies examining school neighbourhood BE features and their relationship to adolescents’ ATS in diverse urbanisation settings. However, previous studies conducted in large urban areas and rural areas reported that adolescents were more likely to use ATS in school neighbourhoods with higher intersection density, higher residential density, and higher walkability score in the United States and Spain [22,23,38,39]. In addition, differences in school neighbourhood BE features may in part explain different ATS rates in urban compared to rural adolescents reported in previous studies [39,40,41,42]. Thus, extending knowledge of school neighbourhood BE features in diverse urbanisation settings is important for tailoring future ATS interventions.

School-level ATS rates among adolescents who lived within 4 km from their school were negatively correlated with school neighbourhood BE residential density, intersection density, and school neighbourhood walkability in both 0.5 km and 1.0 km street-network buffers around individual schools. In contrast, school neighbourhood BE residential density was positively associated with ATS among adolescents in the United States, Belgium, and Finland [15,16,22,23]. Previous studies also reported positive associations [23,38] or no significant associations [21] between adolescents’ ATS rates and school neighbourhood BE intersection density. In addition, one New Zealand study reported no association between school neighbourhood walkability and school-level ATS rates among adolescents who lived within walking distance to school [21]. Possible explanations for the different findings in this study compared to other studies could be the analysis of school-level school neighbourhood BE data. Another explanation could be related to the present study’s exclusion of adolescents who lived beyond cycling distance (4 km) from their school and the inclusion of school locations in diverse urbanisation settings. It is noted that residential density may be an indicator to determine supportive features for walking and to ensure the presence of more people on the street to create a safer social environment along the walking routes to school [23]. Low intersection density along the walking and cycling routes to school may also encourage adolescents to use ATS [38]. Therefore, school neighbourhood BE residential density, intersection density, and neighbourhood walkability should be considered in school settings and school neighbourhood re-development practices.

Adolescents’ perceptions of high traffic volume, and presence of dangerous crossings along the school routes were negatively correlated with school-level ATS. Similar findings related to perceptions of high traffic volume and the presence of dangerous crossings have been previously found among adolescents [11,21]. In addition, an Irish study reported that the rates of ATS increased among adolescents if safe road crossings for walking or cycling to school were available along the routes to school [17]. Consistent with one previous study [21], school-level ATS rates were also negatively correlated with adolescents, and their parents (as reported by adolescents) perceived safety concerns related to walking or cycling to school. Previous studies also found that the rates of ATS decreased if walking or cycling routes to school were perceived as less safe to adolescents and their parents [11,32,43]. However, the present study did not distinguish adolescents’ perceptions of traffic versus personal safety when determining their impression that walking or cycling to school was unsafe. Future ATS interventions should address adolescents’ perceptions of traffic and personal safety concerns related to walking and cycling routes to school.

Both objective measurement of the school neighbourhood BE (including residential density, intersection density, and neighbourhood walkability) and adolescents’ perceptions of safety along the walking and cycling routes to school were found to be significant correlates of school-level ATS rates among adolescents. Our recently published framework related to safe routes to school design also showed that residential density, intersection density, and neighbourhood walkability were important correlates of ATS and should be considered when creating supportive school neighbourhood environment for walking and cycling to school for adolescents [24]. The modification of school neighbourhood BE features when designing interventions (e.g., installing safe road crossings for both pedestrian and cyclists) may encourage adolescents to use ATS.

### 4.1. Implications

The present study contributes to the literature by examining ATS rates at the school-level and their associations with school neighbourhood BE features strictly within walking and/or cycling distance to school, and across different urbanisation settings (i.e., focused on rural areas and smaller urban areas, as well as large urban areas). The present study also contributes to the design of future interventions, particularly related to traffic safety, by identifying whether the school neighbourhood BE features vary, and which features need to be considered to promote ATS among adolescents across different urbanisation settings. School neighbourhood BE intersection density should be considered to promote ATS among adolescents in large and small urban areas because if intersection density increased, the rate of ATS decreased. The design of school routes with few intersections to cross may encourage adolescents to use ATS [23]. Adolescents’ perceptions of safety concerns related to walking and cycling to school in large urban areas should also be considered to increase ATS rates among adolescents. Strategies need to be designed to minimise adolescents’ and parental perceptions of safety concerns. Some of the strategies could include installation of surveillance cameras to minimise the perceived risk of stranger danger and/or crime and installation of traffic lights along the school routes [24]. Due to the negative correlation between school-level ATS rates and adolescents’ traffic safety concerns, the study findings highlighted the importance of minimising adolescents’ concerns related to high traffic volume and dangerous crossings along the school routes, particularly in large urban areas. Construction of safe road crossings and installation of road crossing signs could minimize traffic safety concerns related to both walking and cycling among adolescents using common school routes [44,45].

### 4.2. Study Strengths and Limitations

The major strengths of this study include the involvement of schools located in four diverse urbanisation settings, high rates of school participation (85%) in a single region, GIS assessment of school neighbourhood BE features, and the inclusion of adolescents who lived within a reasonable cycling distance to school (≤4.0 km). However, this study is not without limitations. Despite high school participation rates (85%) and reasonable participation rate of invited adolescents (45%) across both studies, recruitment of adolescents from individual schools varied greatly (range: 17% to 96%) which has effect on validity of school-level data used in our analysis. In this study, three schools (13%) had adolescent recruitment rate of less than 20% study which may have compromised validity of school-level data for those schools. Additional nine schools (39%) had adolescent recruitment rate between 30% and 49%. Additional study limitations include a small number of schools in each urbanisation setting, no major urban centres in the Otago region (therefore, no data from schools for that setting), study participants who lived both within and outside of their school neighbourhoods, assessment of adolescents’ perceptions related to school route rather than the school neighbourhood, survey items not differentiating personal versus traffic safety concerns related to walking and/or cycling to school, and not including other perceived environmental qualities (e.g., building, shops, and neighbourhood safety issues including crime). Finally, due to context-specific factors, the findings of this study have limited generalisability to other regions of New Zealand or internationally. However, that applies to many other studies that are examining BE features and ATS. Future studies should examine the association between school neighbourhood BE features with ATS rates in different geographical areas and with a large sample of schools from a range of urbanisation settings. Future research should also consider differentiating adolescents’ concerns about their personal versus traffic safety along the school routes in school neighbourhood factoring in other perceived BE characteristics.

## 5. Conclusions

School-level ATS rates of adolescents who lived within 4.0 km of their school were negatively correlated with objectively measured school neighbourhood BE, such as residential density, intersection density, and neighbourhood walkability. The school neighbourhood BE intersection density was significantly different between large urban areas, small urban areas, and rural settings. Residential density was also higher in large urban areas and significantly different across all four urbanisation settings within 1.0 km street-network buffer of each school. In addition, adolescents’ perceptions of high traffic volume and dangerous crossings along the school routes and safety concerns related to walking and cycling to school were negatively correlated with ATS. Therefore, future initiatives to encourage ATS should focus on creating ATS-promoting school neighbourhood BE features and minimising adolescents’ traffic and personal safety concerns for walking and cycling to/from school, particularly in large urban areas.

## Figures and Tables

**Table 1:** School characteristics in different urbanisation settings.

	Schools					
	All Schools	Large Urban Area	Medium Urban Areas	Small Urban Areas	Rural Settings	*p*-Value
Number of schools (n)	23	11	3	4	5	
Number of surveyed adolescents per school living within 4 km from school (mean ± SD)	54.7 ± 36.0	62.5 ± 31.9 ^b^	47.7 ± 40.2	87.5 ± 26.3	15.8 ± 9.3 ^a^	0.010
School-level rates of active transport to school (%) (mean ± SD)	48.3 ± 11.7	41.8 ± 10.8 ^b^	43.6 ± 5.2	55.6 ± 9.3	59.5 ± 6.5 ^a^	0.008
Proportion of adolescents living within walking and cycling distances to school						
Within walking distance (≤2.25 km) (%) (school-level mean ± SD)	68.7 ± 23.3	54.2 ± 22.3 ^b^	75.1 ± 9.7	75.8 ±19.5	91.1 ± 11.0 ^a^	0.012
Beyond walking and within cycling distance (>2.25 km to ≤4.0 km) (%) (school-level mean ± SD)	31.3 ± 23.3	45.9 ±22.3 ^b^	24.9 ± 9.7	24.2 ± 19.5	8.9 ± 11.0 ^a^	0.012

^a^*p* < 0.05 versus large urban area, ^b^ *p* < 0.05 versus rural settings.

**Table 2:** Sociodemographic characteristics of adolescent participants.

	Adolescents					
	Total Sample	Large Urban Areas	Medium Urban Areas	Small Urban Areas	Rural Settings	*p*-Value
	(n = 1260)	(n = 688)	(n = 143)	(n = 350)	(n = 79)	
Age (years) (mean ± SD)	15.2 ± 1.4	15.2 ± 1.4	15.3 ± 1.4	15.1 ± 1.3	15.4 ± 1.5	0.190
Male gender (n (%))	549 (43.6)	295 (42.7)	65 (45.5)	156 (44.6)	34 (43.0)	0.784
Ethnicity (n (%))	(n = 1256)	(n = 685)	(n = 143)	(n = 350)	(n = 78)	
New Zealand European	897 (71.4)	475 (69.3)	112 (78.3)	251 (71.7)	59 (75.6)	
Māori	146 (11.6)	78 (11.4)	10 (7.0)	46 (13.1)	12 (15.4)	
Other	213 (17.0)	132 (19.3)	21 (14.7)	53 (15.1)	7 (9.0)	0.060
New Zealand index of deprivation (n (%))	(n = 1225)	(n = 680)	(n = 119)	(n = 347)	(n = 79)	
1 (least deprived)	270 (22.0)	166 (24.4)	29 (24.4)	74 (21.3)	1 (1.3)	
2	286 (23.3)	137 (20.1)	32 (26.9)	89 (25.6)	28 (35.4)	
3	320 (26.1)	153 (22.5)	26 (21.8)	115 (33.1)	26 (32.9)	
4	235 (19.2)	136 (20.0)	22 (18.5)	53 (15.3)	24 (30.4)	
5 (most deprived)	114 (9.3)	88 (12.9)	10 (8.4)	16 (4.6)	0 (0.0)	<0.001
Number of bikes available to use to get to school (n (%))						
None	288 (22.9)	216 (31.4)	21 (14.7)	48 (13.7)	3 (3.8)	
One	253 (20.1)	141 (20.5)	29 (20.3)	62 (17.7)	21 (26.6)	
Two or more	719 (57.1)	331 (48.1)	93 (65.0)	240 (68.6)	55 (69.6)	<0.001
Number of vehicles at home (n (%))						
None	41 (3.3)	35 (5.1)	3 (2.1)	3 (0.9)	0 (0.0)	
One	359 (28.5)	253 (36.8)	25 (17.5)	66 (18.9)	15 (19.0)	
Two or more	860 (68.3)	400 (58.1)	115 (80.4)	281 (80.3)	64 (81.0)	<0.001
Distance to school (km) (mean ± SD)	1.8 ± 1.0	2.0 ± 1.1 ^b,c,d^	1.7 ± 0.8 ^a,d^	1.7 ± 1.0 ^a,d^	1.1 ± 0.9 ^a,b,c^	<0.001

SD = Standard deviation. ^a^ *p* < 0.05 versus large urban area, ^b^ *p* < 0.05 versus medium urban area, ^c^ *p* < 0.05 versus small urban area, ^d^ *p* < 0.05 versus rural settings.

**Table 3:** Objectively measured and perceived built environment (BE) characteristics of the school neighbourhood.

	Total Sample	Large Urban Area	Medium Urban Areas	Small Urban Areas	Rural Settings	*p*-Value
	(23 schools)	(11 schools)	(3 schools)	(4 schools)	(5 schools)	
**Objective measures**						
GIS data: 0.5 km street-network buffer						
Intersection density	47.23 ± 20.71	58.53 ± 24.06	40.41 ± 2.41	31.63 ± 3.54	38.92 ± 13.57	0.071
Residential density	727.60 ± 358.76	948.57 ± 365.48 ^d^	766.27 ± 108.21	567.58 ± 109.87	346.26 ± 127.56 ^a^	0.005
Mixed land use	0.47 ± 0.19	0.42 ± 0.23	0.37 ± 0.32	0.61 ± 0.13	0.52 ± 0.12	0.230
Walkability index	0.00 ± 1.63	0.89 ± 1.81	−0.73 ± 0.54	−0.42 ± 0.59	−1.20 ± 1.20	0.063
GIS data: 1.0 km street−network buffer						
Intersection density	47.37 ± 19.01	60.19 ± 19.35 ^c,d^	41.93 ± 9.30	34.88 ± 5.03 ^a^	32.41 ± 8.94 ^a^	0.008
Residential density	746.79 ± 344.09	989.01 ± 259.35 ^c,d^	819.67 ± 176.69 ^d^	595.41 ± 74.97 ^a^	291.31 ± 125.15 ^a,b^	<0.001
Mixed land use	0.46 ± 0.15	0.42 ± 0.17	0.42 ± 0.08	0.60 ± 0.10	0.46 ± 0.12	0.230
Walkability index	0.00 ± 1.75	1.12 ± 1.45 ^d^	−0.32 ± 1.36	−0.17 ± 0.44	−2.12 ± 1.15 ^a^	0.002
Adolescents’ perceptions of route to school						
There is too much traffic along the route *	1.82 ± 0.41	2.09 ± 0.29 ^d^	1.88 ± 0.13	1.67 ± 0.30	1.30 ± 0.24 ^a^	<0.001
There is one or more dangerous crossings along the route *	1.86 ± 0.34	2.04 ± 0.28 ^d^	1.94 ± 0.14	1.85 ± 0.23	1.40 ± 0.22 ^a^	0.001
Adolescents’ perceptions of walking to school						
It is unsafe to walk to school *	1.42 ± 0.22	1.55 ± 0.20 ^d^	1.39 ± 0.17	1.42 ± 0.14	1.15 ± 0.07 ^a^	0.003
My parents think it is not safe to walk to school *	1.40 ± 0.23	1.51 ± 0.22 ^d^	1.40 ± 0.27	1.36 ± 0.12	1.17 ± 0.15 ^a^	0.042
There are no footpaths along the way *	1.39 ± 0.14	1.35 ± 0.11	1.30 ± 0.07	1.53 ± 0.05	1.43 ± 0.22	0.086
Adolescents’ perceptions of cycling to school						
It is unsafe to cycle to school *	2.02 ± 0.48	2.38 ± 0.31 ^c,d^	2.08 ± 0.37	1.75 ± 0.30 ^a^	1.42 ± 0.19 ^a^	<0.001
My parents think it is not safe to cycle to school *	1.85 ± 0.44	2.16 ± 0.34 ^c,d^	1.88 ± 0.31	1.61 ± 0.20 ^a^	1.33 ± 0.14 ^a^	<0.001
There are no cycle paths along the way *	2.83 ± 0.43	2.93 ± 0.34	2.30 ± 0.14	2.73 ± 0.22	3.02 ± 0.62	0.087

^a^*p* < 0.05 versus large urban area, ^b^ *p* < 0.05 versus medium urban area, ^c^ *p* < 0.05 versus small urban area, ^d^ *p* < 0.05 versus rural settings.* Data collected using 4-point Likert scale (1 = Strongly disagree to 4 = Strongly agree).

**Table 4:** Correlations between school-level rates of active transport to school, objectively measured BE features, and adolescents’ perceptions of school routes.

	School-Level Active Transport to School Rates	0.5 km Street-network Buffer				1.0 km Street-network Buffer			
		Intersection Density	Residential Density	Land Use Mix	Walkability Index	Intersection Density	Residential Density	Land Use Mix	Walkability Index
School-level active transport to school rates		−0.61 **	−0.62 **	0.09 *	−0.69 ***	−0.58 ***	−0.60 **	0.07 *	−0.64 ***
Adolescents’ perceptions of route to school									
There is too much traffic along the route	−0.82 ***	0.57 **	0.63 **	−0.07	0.69 ***	0.67 ***	0.70 ***	−0.02	0.77 ***
There are one or more dangerous crossings along the route	−0.08 ***	0.55 **	0.65 **	−0.05	0.71 ***	0.62 **	0.67 ***	−0.01	0.74 ***
Adolescents’ perceptions of walking to school									
It is unsafe to walk to school	−0.76 ***	0.51 *	0.52 *	0.13	0.71 ***	0.49 *	0.55 **	0.20	0.71 ***
My parents think it is not safe to walk to school	−0.84 ***	0.46 *	0.48 *	0.22	0.71 ***	0.39	0.47 *	0.29	0.66 **
There are no footpaths along the way	0.20	−0.28	−0.37	0.29	−0.22	−0.32	−0.37	0.33	−0.21
Adolescents’ perceptions of cycling to school									
It is unsafe to cycle to school	−0.78 ***	0.51 *	0.54 **	−0.11	0.57 **	0.55 **	0.58 **	−0.06	0.61 **
My parents think it is not safe to cycle to school	−0.82 ***	0.61 **	0.61 **	−0.10	0.69 ***	0.61 **	0.61 **	−0.08	0.65 **
There are no cycle paths along the way	−0.09	0.31	0.12	−0.05	0.23	0.16	−0.02	−0.15	−0.01

Data are reported as r (*p*-values as: * *p* < 0.05; ** *p* < 0.01; *** *p* < 0.001).
